# Economic Cost of US Older Adult Assault Injuries

**DOI:** 10.1001/jamanetworkopen.2024.37644

**Published:** 2024-10-04

**Authors:** Cora Peterson, Tadesse Haileyesus, Jeffrey H. Herbst, Melissa S. Gerald, Curtis Florence

**Affiliations:** 1 National Center for Injury Prevention and Control, Centers for Disease Control and Prevention, Atlanta, Georgia; 2 National Institute on Aging, National Institutes of Health, Bethesda, Maryland

## Abstract

This economic evaluation uses nationwide public health data to evaluate incidence and economic costs of homicides and nonfatal assault injuries among US adults aged 60 years or older.

## Introduction

In 2022, crime surveys indicated US older adults experienced 59 000 aggravated and 366 000 simple nonfatal assaults; approximately half of aggravated assaults and one-third of simple assaults were perpetrated by intimate partners, other relatives, or acquaintances.^1^ This is double to triple the corresponding rate point estimates per 100 000 population of a decade prior (1.0 [95% CI, 0.42-1.66] in 2022 vs 0.5 [95% CI, 0.06-0.99] in 2013 for aggravated assaults; 6.5 [95% CI, 4.66-8.26] in 2022 vs 2.1 [95% CI, 0.94-3.23] in 2013 for simple assaults) and exceeds the all-ages rate point differences (aggravated assaults: 45%; simple assaults: −13%). We used national public health surveillance data to report the incidence and economic cost of older adult homicides and emergency department (ED)–treated nonfatal assault injuries.

## Methods

This cross-sectional study of the US population followed STROBE and CHEERS reporting guidelines and did not require institutional review board approval or informed consent per 45 CFR §46. In June 2024, the Centers for Disease Control and Prevention Web-Based Injury Statistics Query and Reporting System Cost of Injury provided the number of homicides and nonfatal assault injury ED visits by sex among people aged 60 years or older and the estimated cost of associated medical spending, lost work productivity, reduced quality of life from injury morbidity, and avoidable mortality using the value of statistical life (VSL) from 2015 to 2022.^2^ The data source uses the societal perspective; undiscounted, inflation-adjusted constant US dollars; and for nonfatal injuries, a 1-year period over which costs are assessed (eAppendix in Supplement 1). Data were analyzed using Microsoft Excel, version 2402.

## Results

**Figure 1.**
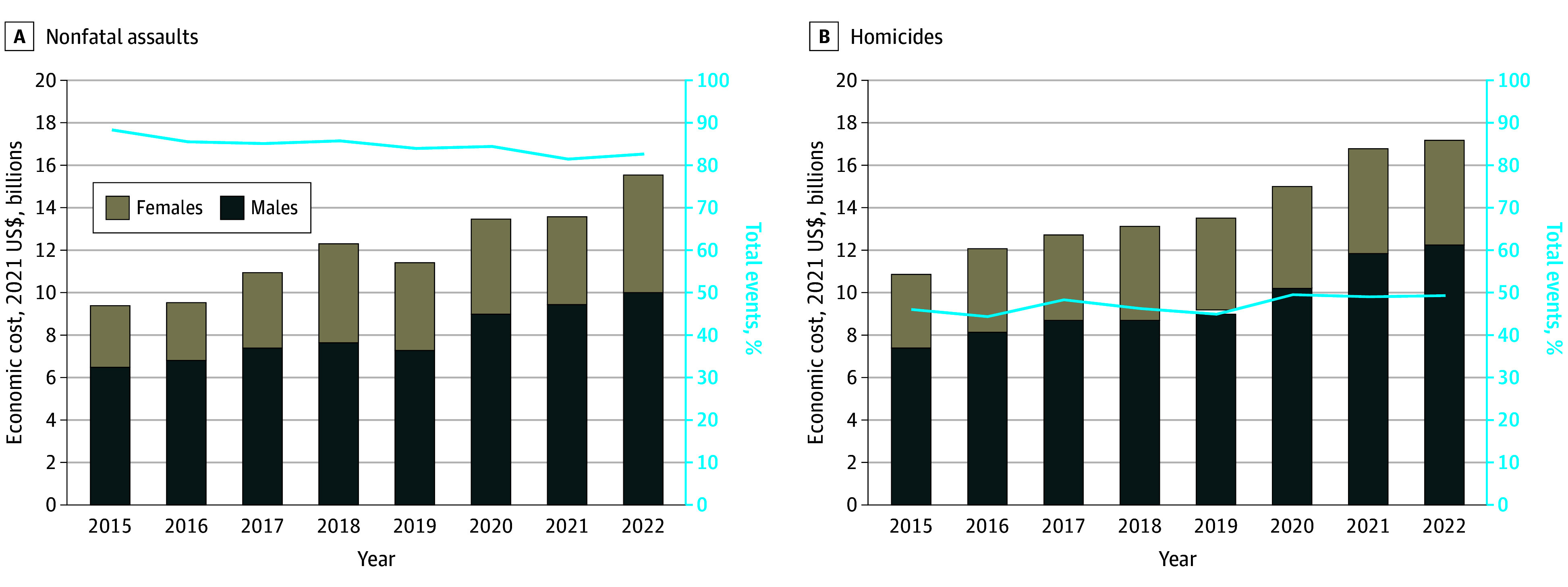
Annual Economic Cost of Homicides and Emergency Department–Treated Nonfatal Assaults of US Adults Aged 60 Years or Older, 2015-2022 Data are from the Centers for Disease Control and Prevention Web-Based Injury Statistics Query and Reporting System, June 2024. Annual mean (SD) cost of nonfatal assaults was $12.0 billion ($2.1 billion) and of homicides was $13.9 billion ($2.2 billion).

**Figure 2.**
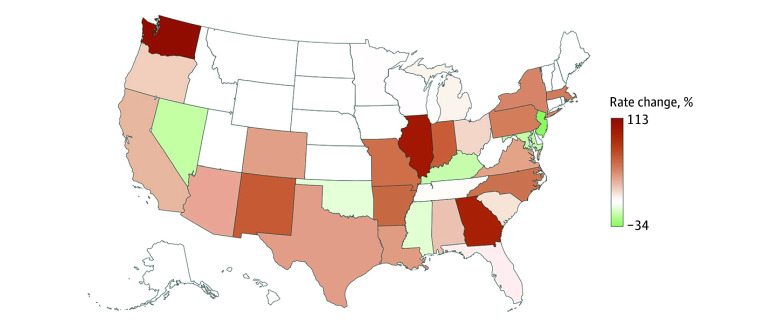
Age-Adjusted Homicide Rate Change Among US Adults Aged 60 Years or Older, 2015-2022 Data are from the Centers for Disease Control and Prevention Web-Based Injury Statistics Query and Reporting System, June 2024. White indicates the rate change was not calculable for the period.

The nationwide annual mean (SD) estimated economic cost of older adult nonfatal assaults and homicides was $25.9 billion ($4.9 billion) from 2015 to 2022, peaking at $32.7 billion in 2022 (Figure 1). In 2022, there were 91 028 (95% CI, 68 099-113 957) ED visits for nonfatal assault injuries (35% females; 65% males) and 2221 homicides (33% female; 67% males); age-adjusted rate point estimates per 100 000 population were notably higher in 2022 compared with 2015 (ED visits: 108.45 vs 82.68; difference, 31%; homicides: 2.74 vs 2.18; difference, 26%). The mean (SD) annual cost was $1.4 billion ($286 million) in medical spending, $179 million ($37 million) in work loss, and $24.3 billion ($4.0 billion) in lost healthy life-years. The average cost per older adult homicide was $13 998 in medical spending and $8 million in VSL and per nonfatal ED-treated assault was $18 498 in medical spending, $2382 in work loss, and $139 298 in reduced quality of life over the following 1 year. Approximately half (44%-55% annually) of older adult homicides resulted from firearm injuries, and most nonfatal assault injuries were from being struck by or against (81%-88% of ED visits annually) (Figure 1). The nationwide homicide rate changed by −4% (2018-2019) to 10% (2019-2020) annually, and by 2022, the homicide rate was at least 50% higher in Washington, Illinois, Georgia, New Mexico, Indiana, Arkansas, Missouri, and North Carolina compared with 2015 (Figure 2).

## Discussion

This study found that the economic cost of older adult violent injuries was substantial. The estimated $122 billion annual cost of youth violence (age 10-24 years) is higher due to more ED visits and homicides.^3^ The study data source is limited by not including criminal justice costs or perpetrator type.

Violence toward older adults is preventable. However, successful prevention depends on use of strategies that target risk factors for older adults and perpetrators, including poor mental health, substance use disorder, and social isolation.^4,5,6^ Prevention and effective violence-related interventions require tailored approaches to address the unique needs of older adults with cognitive and physical impairments and across diverse communities and institutional settings as well as a comprehensive, multidisciplinary team effort involving professionals from health care, social services, law enforcement, and legal sectors.

## Notes

Supplement 1. **eAppendix.** WISQARS Cost of Injury Data Summary

Supplement 2.
Data Sharing Statement
