# Supplementary material for: Economic Cost of US Older Adult Assault Injuries
Source: JAMA Netw Open. 2024 Oct 4;7(10):e2437644. doi: 10.1001/jamanetworkopen.2024.37644 (PMC11452810; doi:10.1001/jamanetworkopen.2024.37644)
Supplement: Supplement 1. — eAppendix. WISQARS Cost of Injury Data Summary [file jamanetwopen-e2437644-s001.pdf]

Supplementary Online Content

Peterson C, Haileyesus T, Herbst JH, Gerald MS, Florence C. Economic cost of US older adult assault injuries. *JAMA Netw Open*. 2024;7(10):e2437644.
doi:10.1001/jamanetworkopen.2024.37644

eAppendix. WISQARS Cost of Injury Data Summary

This supplementary material has been provided by the authors to give readers additional information about their work.

© 2024 Peterson C et al. *JAMA Network Open*.

eAppendix. WISQARS Cost of Injury data summary[1]

- Homicides are from the National Center for Health Statistics[2] and ED visits are from the National Electronic Injury Surveillance System-All Injury Program, a nationally representative probability sample of hospitals.[3] Available data years were 2015-2022 at study time.
- One-year attributable medical spending—including mental health care—and lost work unit costs due to nonfatal injuries by mechanism (e.g., cut/pierce) and intent (e.g., unintentional) are based on analysis of the 2014-5 National Inpatient Sample and National Emergency Department Sample (national estimates of community hospital encounters) and MarketScan Commercial Claims and Encounters Databases (medical payments to providers from approximately 350 health insurance payers—large employers, health plans, and public organizations—and insurance enrollees' work absences from some employers).[4, 5]
  - Inflation to current US dollars uses Bureau of Labor Statistics Table 2.5.4. Price Indexes for Personal Consumption Expenditures by Function / Health expenditures.
  - An average work loss estimate is applied to all ED visits, which assumes non-working injured people (e.g., older adults) require care from a working person.
- The monetary value of injury morbidity and mortality in WISQARS uses quality of life loss estimates for injuries and recommended methods for value of statistical life (VSL), a monetary estimate of the collective value placed on mortality risk reduction as derived in research studies through revealed preferences (e.g., observed wage differences for dangerous occupations) or stated preferences from surveys of individuals' willingness to pay for mortality risk reduction.[6-8] Data source VSL varies by decedent age (in 2021 US dollars: 18-65 years, $11.6 million and descending from $6.5 million [66 years] to US$450,000 [⩾100 years], adjusted for older adults' decreasing general life expectancy, baseline quality of life, and discounted 3% to present value) and each quality-adjusted life year loss was correspondingly valued at $600,000.[1, 8] The relationship between VSL and age is likely more complex than is applied in the data source.
- Changes in the average costs (medical, work loss, VSL, quality of life) of injuries by type due to inflation are minimal compared to the changes resulting from different injury counts year to year. Annual distributions of injury mechanism and injured person age modestly affect cost estimates due to the use of mechanism- and intent-specific medical spending and work loss among injured individuals and VSL that decreases with age.

---

1. Peterson C, Rice KL, Williams DD, Thomas R. WISQARS Cost of Injury for public health research and practice. *Inj Prev.* 2023;29(2):150-157. https://doi.org/10.1136/ip-2022-044708.
2. CDC National Center for Health Statistics. Mortality Statistics. 2023 [cited 2023 July]; Available from: https://www.cdc.gov/nchs/nvss/deaths.htm
3. ISPCR. National Electronic Injury Surveillance System (NEISS) Series. 2023; Available from: https://www.icpsr.umich.edu/web/ICPSR/series/198
4. Peterson C, Xu L, Barnett SBL. Average lost work productivity due to non-fatal injuries by type in the USA. *Inj Prev.* 2021;27(2):111-117. https://doi.org/10.1136/injuryprev-2019-043607.
5. Peterson C, Xu L, Florence C. Average medical cost of fatal and non-fatal injuries by type in the USA. *Inj Prev.* 2021;27(1):24-33. https://doi.org/10.1136/injuryprev-2019-043544.
6. U.S. Department of Health and Human Services Office of the Assistant Secretary for Planning and Evaluation. Guidelines for regulatory impact analysis, Appendix D: Updating Value per Statistical Life (VSL) Estimates for Inflation and Changes in Real Income. 2021; Available from: https://aspe.hhs.gov/reports/updating-vsl-estimates
7. Lawrence B, Miller T. Quality of life loss estimation methods for the WISQARS Cost of Injury Module. Calverton, MD: Pacific Institute for Research and Evaluation; 2020.
8. U.S. Department of Health and Human Services Office of the Assistant Secretary for Planning and Evaluation. Guidelines for regulatory impact analysis. Washington, DC: U.S. Department of Health and Human Services; 2016.

© 2024 Peterson C et al. *JAMA Network Open*.
