# Supplementary material for: Economic Cost of US Older Adult Assault Injuries
Source: JAMA Netw Open. 2024 Oct 4;7(10):e2437644. doi: 10.1001/jamanetworkopen.2024.37644 (PMC11452810; doi:10.1001/jamanetworkopen.2024.37644)
Supplement: Supplement 2. — Data Sharing Statement [file jamanetwopen-e2437644-s002.pdf]

Data Sharing Statement

Peterson. Economic Cost of US Older Adult Assault Injuries. *JAMA Netw Open*. Published October 04, 2024. doi:10.1001/jamanetworkopen.2024.37644

Data
Data available: Yes
Data types: Data (not involving human participants)
How to access data: CDC Web-based Injury Statistics Query and Reporting System, https://wisqars.cdc.gov
When available: With publication

Supporting Documents
Document types: None

Additional Information
Who can access the data: Anyone requesting the data
Types of analyses: Any purpose
Mechanisms of data availability: Without investigator support
Any additional restrictions: None
